# Low geriatric nutritional risk index as a poor prognostic biomarker for immune checkpoint inhibitor treatment in solid cancer

**DOI:** 10.3389/fnut.2023.1286583

**Published:** 2023-11-01

**Authors:** Lilong Zhang, Kunpeng Wang, Tianrui Kuang, Wenhong Deng, Peng Hu, Weixing Wang

**Affiliations:** ^1^Department of General Surgery, Renmin Hospital of Wuhan University, Wuhan, Hubei Province, China; ^2^Department of Emergency, Renmin Hospital of Wuhan University, Wuhan, China

**Keywords:** biomarker, cancers, geriatric nutritional risk index, immune checkpoint inhibitors, outcomes

## Abstract

**Objective:**

In this investigation, we focused on the geriatric nutritional risk index (GNRI), a comprehensive metric that takes into account the patient’s ideal weight, actual weight, and serum albumin levels to measure malnutrition. Our primary objective was to examine the predictive value of GNRI-defined malnutrition in determining the response to immunotherapy among cancer patients.

**Methods:**

Relevant articles for this study were systematically searched in PubMed, the Cochrane Library, EMBASE, and Google Scholar up to July 2023. Our analysis evaluated overall survival (OS), progression-free survival (PFS), objective response rate (ORR), and disease control rate (DCR) as clinical outcomes.

**Results:**

This analysis comprised a total of eleven articles encompassing 1,417 patients. The pooled results revealed that cancer patients with low GNRI levels exhibited shorter OS (HR: 2.64, 95% CI: 2.08–3.36, *p* < 0.001) and PFS (HR: 1.87, 95% CI: 1.46–2.41, *p* < 0.001), and lower ORR (OR: 0.46, 95% CI: 0.33–0.65, *p* < 0.001) and DCR (OR: 0.42, 95% CI: 0.29–0.61, *p* < 0.001). Sensitivity analyses confirmed that the above results were stable. Egger’s and Begg’s tests revealed that there was no publication bias in the above results.

**Conclusion:**

Our results imply that the GNRI is a useful predictor of immunotherapy response in cancer patients.

## 1. Introduction

With the rising use of immune checkpoint inhibitors (ICIs) in tumor treatment, there has been significant research on identifying novel biomarkers that can effectively predict the response to ICI therapy (1–3). Traditionally, PD-L1 expression in tumor tissue has been considered a prominent marker for PD-(L)1 therapy due to its mechanistic relevance (1, 4). Additionally, the tumor mutational burden, which reflects the total number of somatic mutations, has also emerged as a predictive sign for ICIs and has been authorized as a companion diagnostic test (1, 5, 6). In contrast to oncogenic driver mutations for targeted therapy, these biomarkers are insufficient to identify ICI responders. For instance, even individuals with NSCLC and strong PD-L1 expression only exhibit an ORR of 44.8% when treated with pembrolizumab (7). Conversely, patients with low PD-L1 expression may also benefit from ICIs (8–10). This discrepancy indicates that tissue-based approaches alone are insufficient for predicting ICI therapy outcomes. ICIs stimulate antitumor responses through immune cells, in contrast to targeted treatments, which have direct antitumor effects on tumor cells. Thus, assessing host factors in addition to tumor characteristics may provide crucial information for accurately predicting the efficacy of ICIs.

It is well known that nutritional status is linked to immune function and influences the clinical consequences of various diseases, including cancer (11–13). The Geriatric Nutritional Risk Index (GNRI) is a simple and convenient nutritional assessment tool that utilizes serum albumin levels and the ratio of actual to ideal body weight (14–16). It has been associated with mortality in elderly patients as well as those with cardiovascular disease and various cancers (17–20). In the field of cancer treatment, GNRI has been related to survival following chemotherapy, surgery, or chemoradiotherapy in various malignancies (21–23). Additionally, although GNRI was initially created for older people, it can be used for younger populations as well (24–26).

However, the effectiveness of the GNRI in predicting the efficacy of ICI treatment remains a subject of debate. Therefore, the purpose of our study was to comprehensively assess the prediction value of GNRI in ICI-treated cancer patients. The outcomes of this research will contribute to the development of effective treatment strategies that enable precise and cost-effective therapies with minimal adverse effects.

## 2. Methods

### 2.1. Strategies for literature search

The current study followed the guidelines outlined in the PRISMA statement (27). On July 1, 2023, a comprehensive literature search was conducted using PubMed, EMBASE, and the Cochrane Library. Supplementary Table S1 provides a comprehensive description of the search strategies. In addition, Google Scholar was used to research grey literature, and the reference lists of eligible studies were manually screened.

### 2.2. Criteria for inclusion and exclusion

Strict inclusion criteria were applied in this study, focusing on articles that evaluated the prognostic value of GNRI in cancer patients undergoing ICI treatment. Only articles reporting relevant outcomes such as OS, PFS, ORR, and DCR were included. Conference abstracts were excluded from the analysis. We chose the trials with the most thorough data and robust methodology when studies had overlapping patients (28).

### 2.3. Data extraction and quality assessment

A comprehensive range of information was extracted from the selected articles, including author names, study design, duration and location of the study, drugs used for treatment, cancer type, sample size, patient demographics (age and gender), and outcomes. In cases where both univariate and multivariate analyses were conducted, greater emphasis was placed on the data from multivariate analyses. The quality of observational studies was assessed using the Newcastle-Ottawa Scale (NOS), with literature scoring 6 or above considered high-quality (29).

### 2.4. Statistical methods

For statistical analysis, Stata 15.0 software was used. We used the chi-squared test to determine heterogeneity, and when the *p*-value was less than 0.1, we selected a random-effects model; otherwise, we selected a fixed-effects model. To calculate publication bias, we used the Egger’s and Begg’s tests. We also conducted a sensitivity analysis, eliminating each study separately, to assess the validity of the results.

## 3. Results

### 3.1. Characteristics of studies

**Figure 1:**
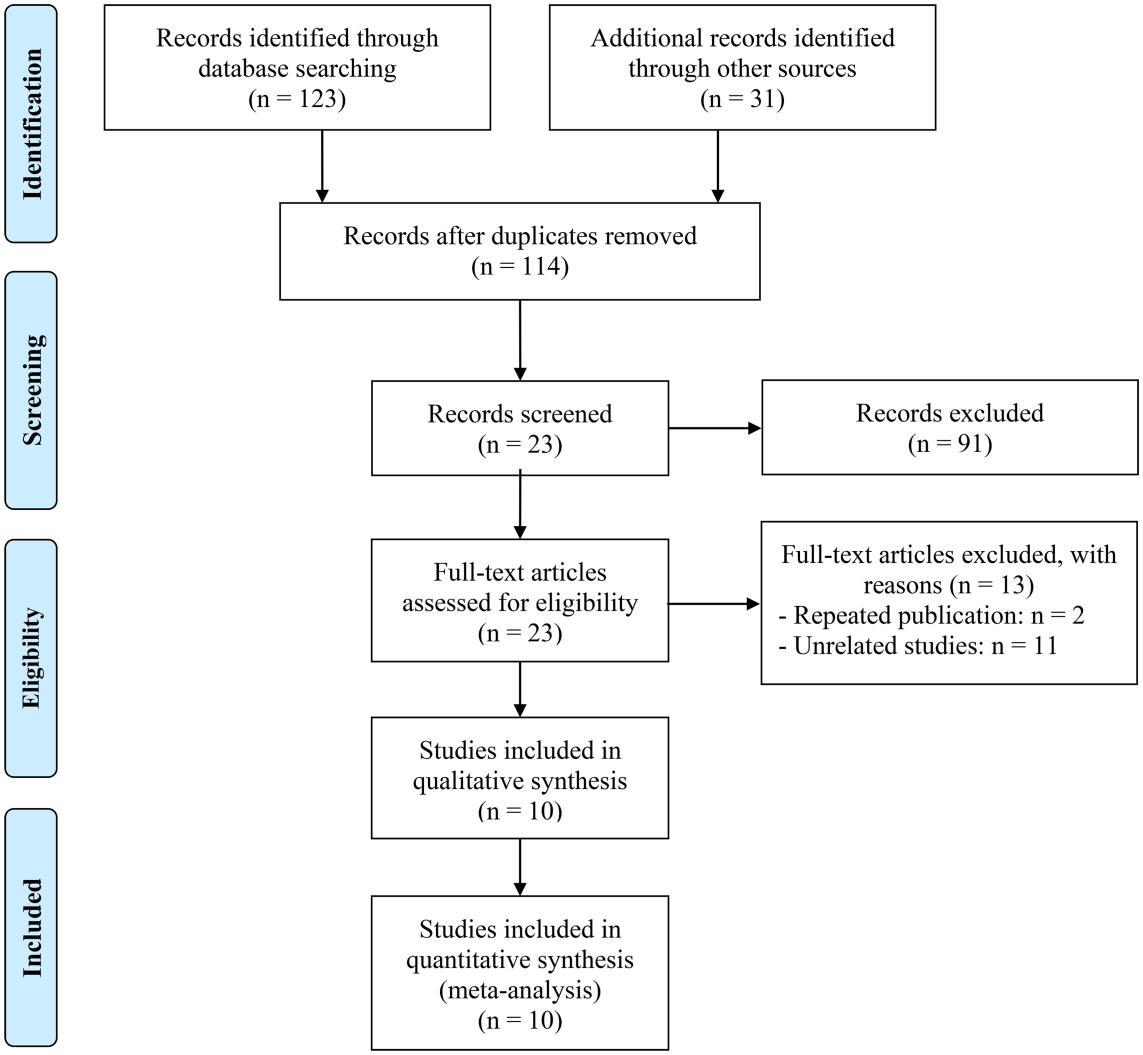
The flow diagram for identifying eligible studies.

We were left with 23 publications to evaluate in full text after removing duplicates and analyzing titles and abstracts. A total of eleven studies with 1,417 patients were included in the final analysis (26, 30–39). Figure 1 uses a PRISMA flowchart to show the research selection process. Table 1 lists all of the specific characteristics of the accepted studies. Using the NOS, the risk of bias in each of the included studies was evaluated; scores between 6 and 8 denote a low risk of bias.

**Table 1:** Main characteristics of the studies included.

Study	Study design	Study period	Study region	ICI treatment	Cancer type	Sample size	Age (years)	Gender (male/female)	Outcome
Zheng et al. (38)	*R*	03/2020–06/2022	China	Tislelizumab	CC	115	54 (32–70)^a^	0/115	PFS, ORR
Liu et al. (39)	*R*	01/2018–12/2021	China	ICIs treatment	HCC	101	57.8 ± 9.29	83/18	OS, PFS
Tanaka et al. (37)	*R*	04/2017–12/2020	Japan	Nivolumab	HNSCC	42	60.5 (26–81)^d^	36/6	OS, ORR, DCR
Haas et al. (32)	*R*	2016–2021	Austria	Nivolumab or pembrolizumab	HNSCC	162	65 (28–85)^a^	115/47	OS, PFS, ORR, DCR
Hiraoka et al. (33)	*R*	09/2020–07/2022	Japan	Atezolizumab + Bevacizumab	HCC	525	74 (68–80)^b^	420/105	ORR, DCR
Fujiwara et al. (31)	*R*	09/2013–08/2020	Japan	Nivolumab	RCC	56	62 (56–69)^b^	42/14	OS, PFS, ORR
Karayama et al. (35)	*P*	07/2016–12/2018	Japan	Nivolumab	NSCLC	158	69 (40–83)^a^	129/29	OS, PFS
Isobe et al. (34)	*R*	07/2009–02/2021	Japan	Pembrolizumab	UC	94	72 (47–85)^a^	77/17	OS
Sonehara et al. (26)	*R*	02/2016–10/2020	Japan	Nivolumab, Pembrolizumab, Atezolizumab	NSCLC	85	39/46^c^	68/17	OS, PFS, ORR, DCR
Shimizu et al. (36)	*R*	12/2017–08/2019	Japan	Pembrolizumab	UC	27	73 (52–82)^a^	23/4	OS, PFS
Etani et al. (30)	*R*	01/2018–10/2019	Japan	Pembrolizumab	UC	52	71 (46–84)^a^	43/9	ORR

a Medians (ranges).

b Medians (interquartile range).

c ≥ 70 vs. < 70.

d Mean(ranges); *R*, retrospective study; *P*, prospective study; CC, cervical cancer; HNSCC, head and neck squamous cell carcinoma; HCC, hepatocellular carcinoma; RCC, renal cell carcinoma; NSCLC, non-small cell lung cancer; UC, urothelial cancer; ICIs, immune checkpoint inhibitors; OS, overall survival; PFS, progression-free survival; ORR, objective response rate; DCR, disease control rate.

Three studies of urothelial cancer, two studies of non-small cell lung cancer, hepatocellular carcinoma, and head and neck squamous cell carcinoma were included in this study. Most of the studies were retrospective designs implemented in Japan. The timeframe for publication of the article is 2020–2023.

### 3.2. Baseline GNRI levels and OS

**Figure 2:**
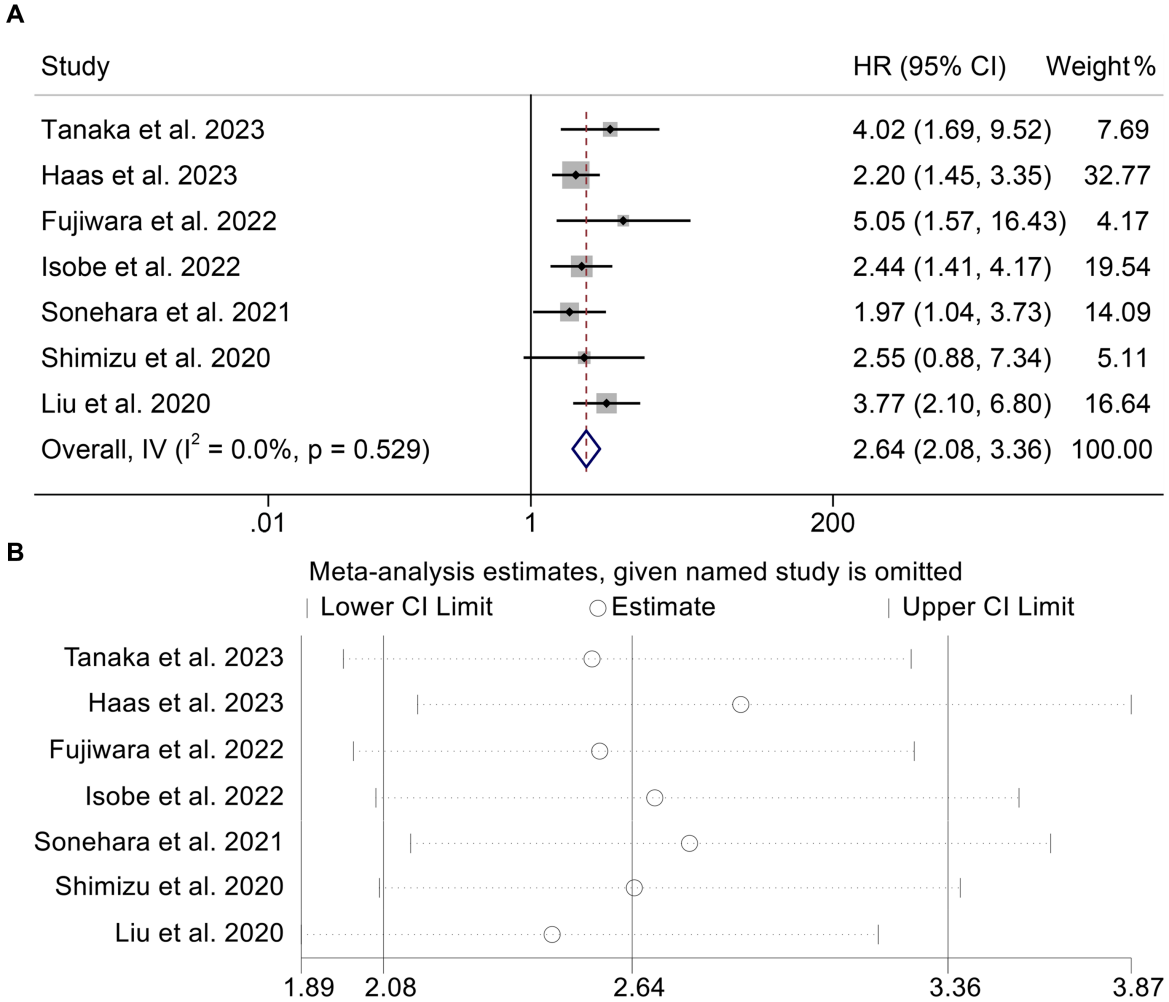
Forest plots of the relationship between geriatric nutritional risk index and overall survival **(A)**. Sensitivity analysis of the association between geriatric nutritional risk index and overall survival **(B)**. HR, hazard ratio; CL, confidence interval.

We sought to investigate the relationship between GNRI levels (as a binary categorical variable) and OS in patients with solid tumors receiving ICI by the analysis of data from seven studies involving 567 participants. We found patients with low GNRI had a shorter OS compared to patients with high GNRI (HR: 2.64, 95% CI: 2.08–3.36, *p* < 0.001, Figure 2A). The analysis above used a fixed effects model because there was no significant heterogeneity (*I*^2^ = 0%, *p* = 0.529). No publication bias in the aforementioned results was verified by Begg’s and Egger’s tests (Begg’s: *p* = 0.230, Egger’s: *p* = 0.174). By gradually removing each study and analyzing the effects on the combined findings, we carried out a sensitivity analysis to assess the reliability of our findings. Our findings showed that the pooled HR was not significantly affected by the deletion of any particular study, ranging from 2.46 [95% CI: 1.89–3.20, after removing Liu et al. (39)] to 2.89 [95% CI: 2.16–3.87, after removing Haas et al. (32), Figure 2B].

**Figure 3:**
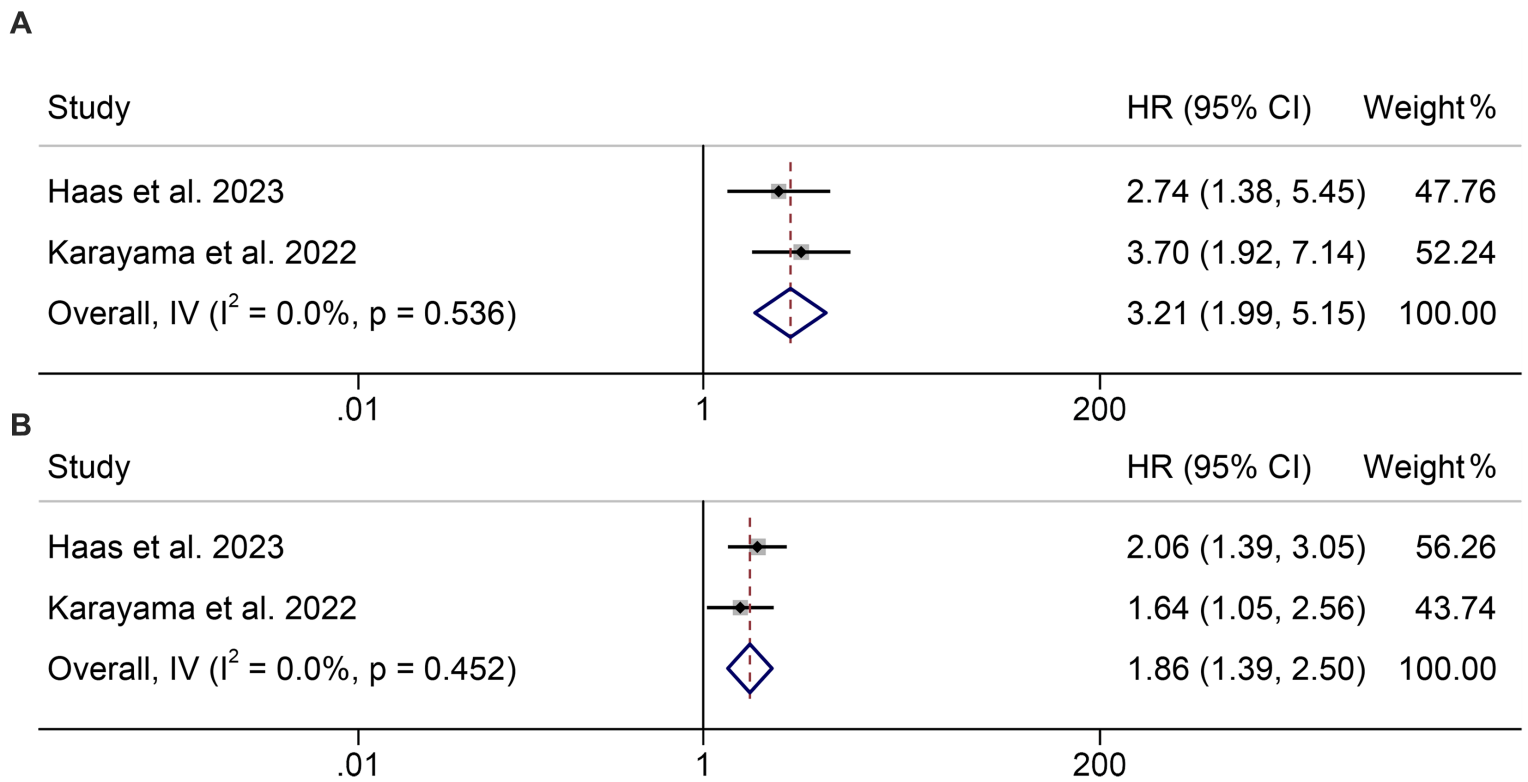
Forest plots of the relationship between geriatric nutritional risk index and overall survival. **(A)** <82 vs. >98; **(B)** 98–82 vs. >98. HR, hazard ratio; CL, confidence interval.

In addition, two studies with 320 patients analyzed the GNRI as a triple categorical variable based on cut-off values of 98 and 82. We found that the lower the GNRI, the shorter the OS of cancer patients (<82 vs. >98, HR: 3.21, 95% CI: 1.99–5.15, *p* < 0.001, Figure 3A; 98–82 vs. >98, HR: 1.86, 95% CI: 1.39–2.50, *p* < 0.001, Figure 3B).

### 3.3. Baseline GNRI levels and PFS

**Figure 4:**
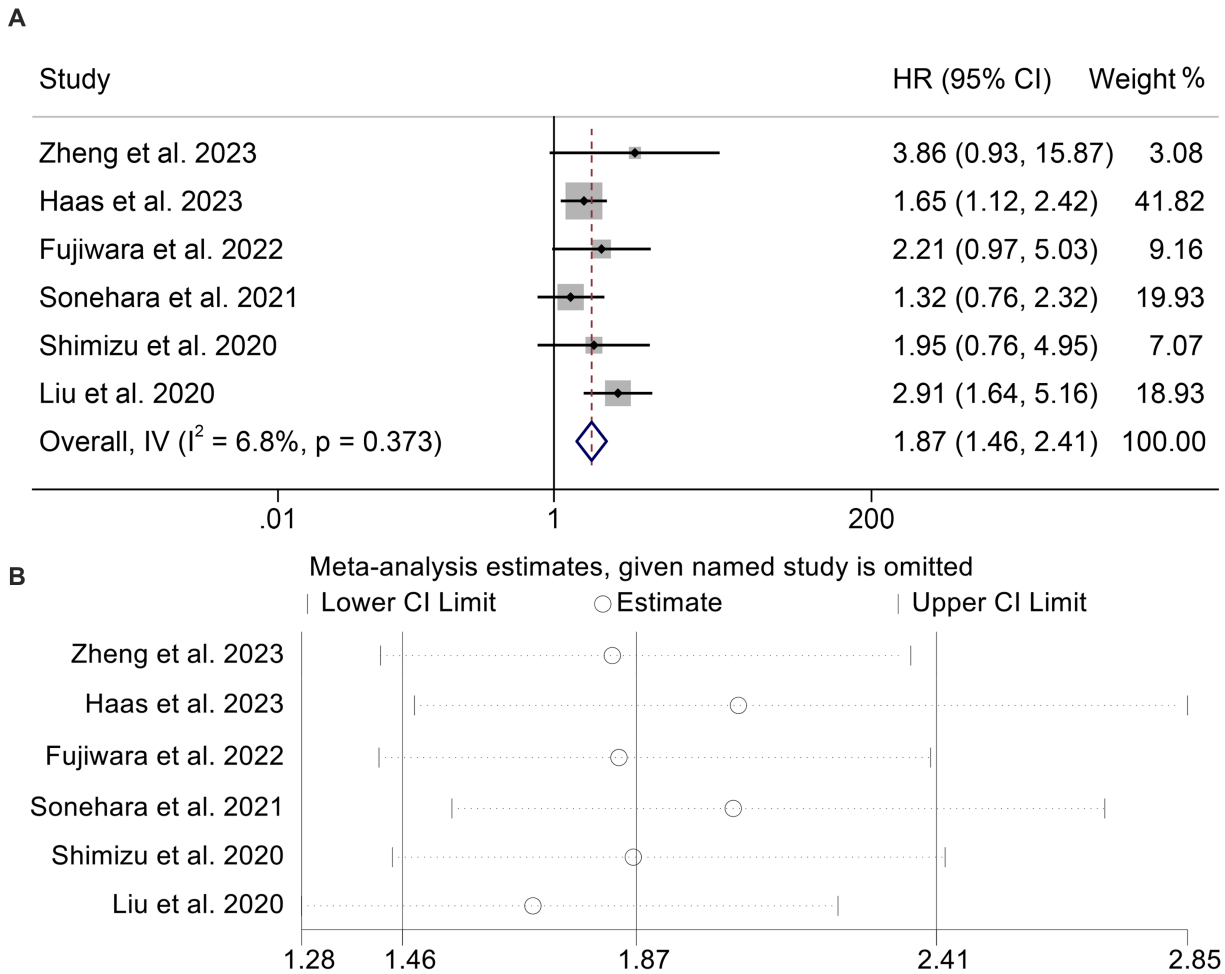
Forest plots of the relationship between geriatric nutritional risk index and progression-free survival **(A)**. Sensitivity analysis of the association between geriatric nutritional risk index and progression-free survival **(B)**. HR, hazard ratio; CL, confidence interval.

To determine the connection between GNRI levels and PFS in cancer patients receiving ICIs, we analyzed six studies involving 541 individuals. The results indicated that patients with low GNRI had a higher risk of progression (HR: 1.87, 95% CI: 1.46–2.41, *p* < 0.001, Figure 4A) than those with high GNRI. Because there was no significant heterogeneity (*I*^2^ = 6.8%, *p* = 0.373), the analysis presented above utilized a fixed effects model. Notably, no publication bias was found using the Begg’s and Egger’s tests (Begg’s: *p* = 0.452, Egger’s: *p* = 0.294). According to the findings of the sensitivity analysis, leaving out any of the studies had no significant effect on the pooled HR (Figure 4B).

### 3.4. Baseline GNRI levels and ORR

**Figure 5:**
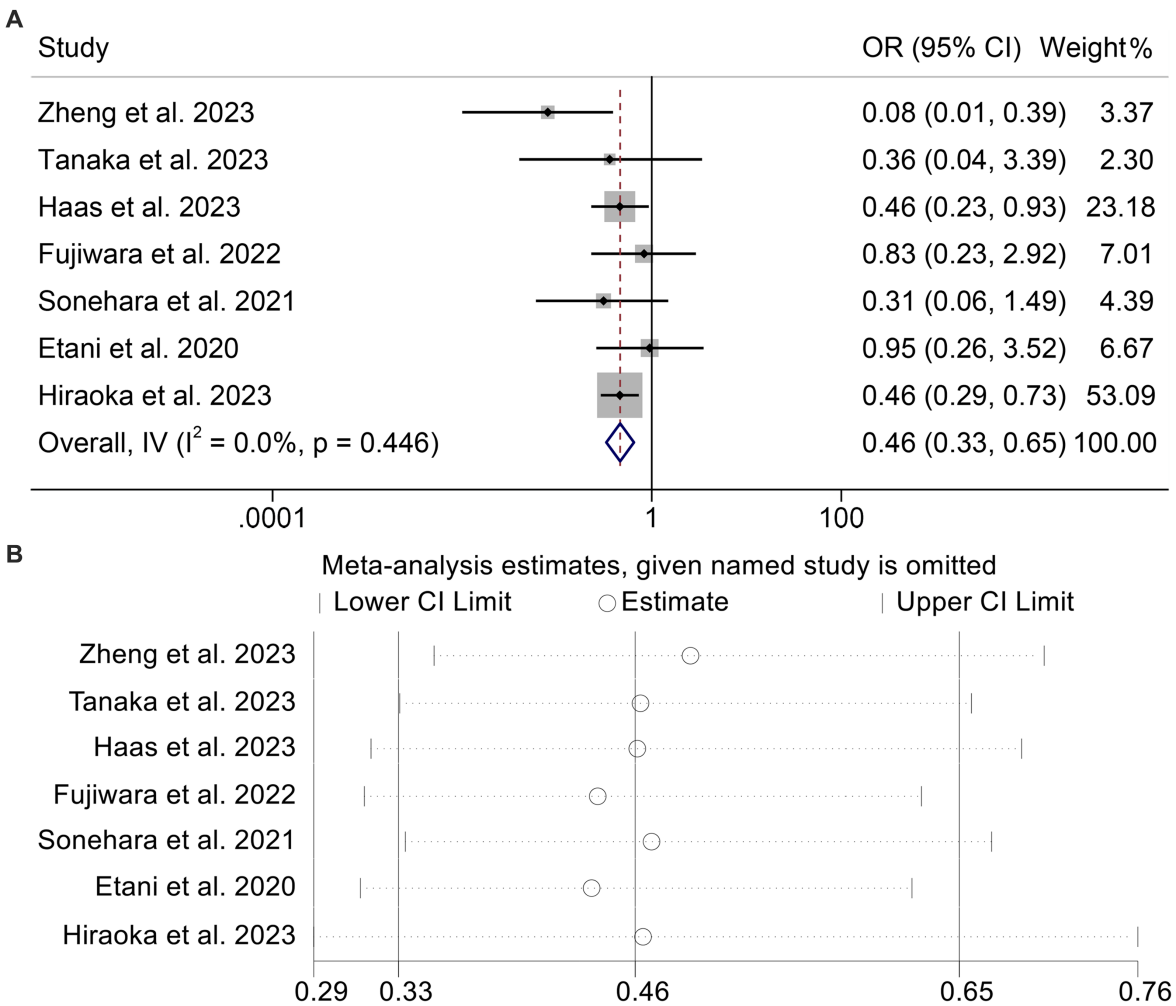
Forest plots of the relationship between geriatric nutritional risk index and objective response rate **(A)**. Sensitivity analysis of the association between geriatric nutritional risk index and objective response rate **(B)**. OR, odds ratio; CL, confidence interval.

Using data from seven studies with a total of 1,037 participants, we analyzed the link between GNRI levels and ORR in cancer patients receiving ICI. Patients with low GNRI had lower ORR than patients with high GNRI (OR: 0.46, 95% CI: 0.33–0.65, *p* < 0.001, Figure 5A). Because there was no significant heterogeneity (*I*^2^ = 0%, *p* = 0.446), a fixed effects model was used in the analysis (*I*^2^ = 0%, *p* = 0.446). Begg’s and Egger’s tests showed no evidence of publication bias in the results mentioned above (Begg’s: *p* = 0.548, Egger’s: *p* = 0.656). We performed a sensitivity analysis to evaluate the stability of our results by gradually deleting each study and examining the implications for the overall findings. Our results showed that the pooled HR was not significantly impacted by the deletion of any individual research (Figure 5B).

### 3.5. Baseline GNRI levels and DCR

**Figure 6:**
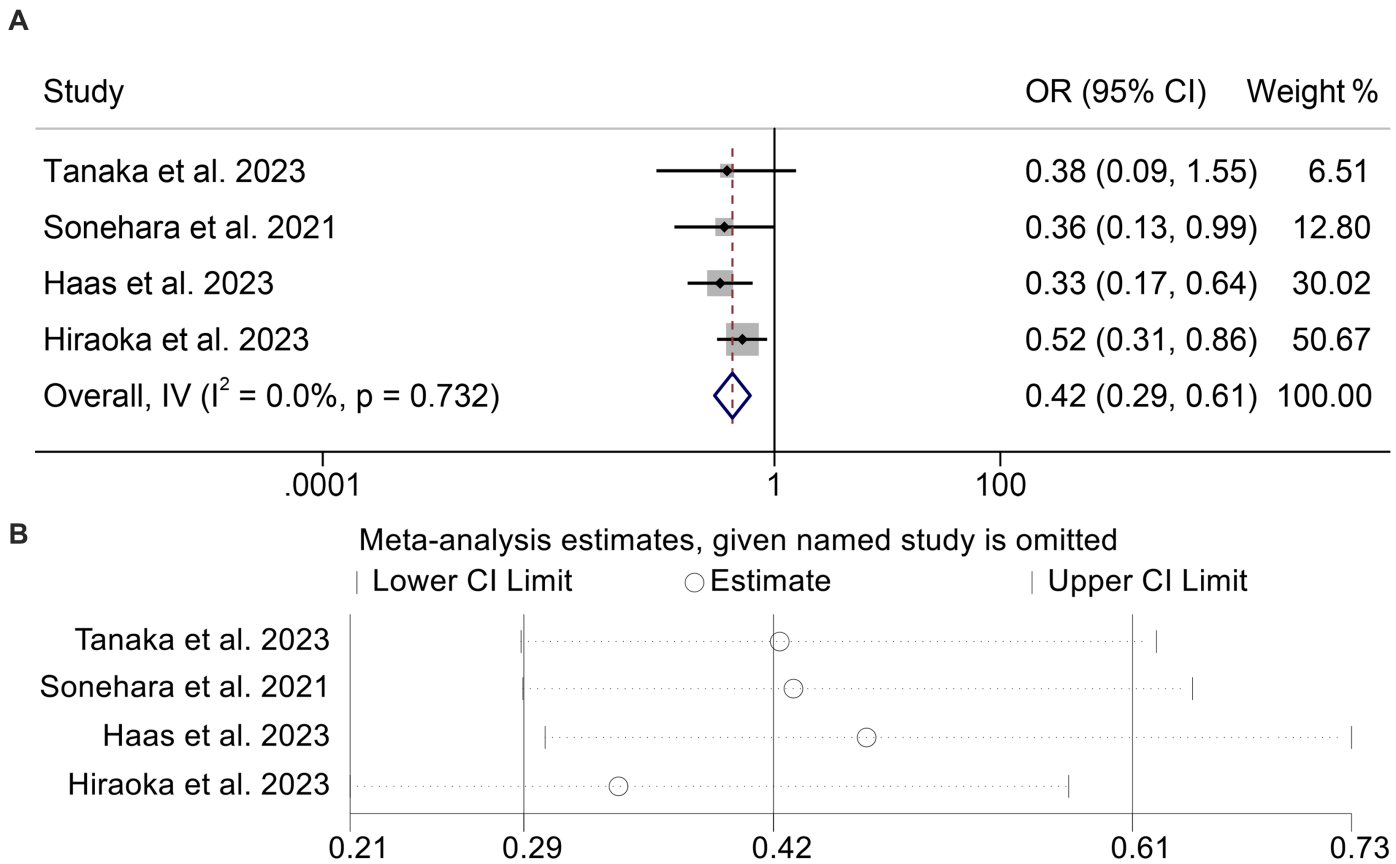
Forest plots of the relationship between geriatric nutritional risk index and disease control rate **(A)**. Sensitivity analysis of the association between geriatric nutritional risk index and disease control rate **(B)**. OR, odds ratio; CL, confidence interval.

We then combined four studies with 814 individuals to investigate the relationship between GNRI levels and DCR in cancer patients. We used a fixed-effect model for our analysis because, as shown in Figure 6A (*I*^2^ = 0.0%, *p* = 0.732), there was no discernible heterogeneity in the results. Patients with low GNRI had a lower DCR than those with high GNRI (OR: 0.42, 95% CI: 0.29–0.61, *p* < 0.001, Figure 6A). No significant publication bias was discovered in the analysis (Begg’s: *p* = 1.000; Egger’s: *p* = 0.467). Sensitivity analyses confirmed no significant effect on the pooled results after deleting any of the studies (Figure 6B).

## 4. Discussion

The aim of our study was to examine the predictive value of GNRI in ICI-treated cancer patients. We found a robust correlation between low GNRI levels and poorer OS and PFS, as well as a lower ORR and DCR. GNRI can be measured cost-effectively, readily, and noninvasively to evaluate nutritional status. Our data suggested that the potential utility of GNRI in predicting the effectiveness of ICI therapy is worth considering.

Malnutrition is a prevalent issue affecting a considerable proportion of patients with advanced diseases, ranging from 30 to 85% (40). This complex condition encompasses reduced protein reserves, caloric depletion, and compromised immune defenses (40, 41). Despite the absence of defined criteria for malnutrition in cancer patients, various nutritional screening tools are currently used to estimate the outcomes of hemodialysis or the prognosis of patients with tumors or infections (42, 43). One well-established screening tool is the subjective global assessment, which has been validated and widely utilized for screening purposes. However, its subjective nature requires examiners to undergo extensive training to ensure consistent and reliable results, given the complexity of the assessment process. For elderly patients, malnutrition assessment has commonly relied on tools such as the Mini-Nutritional Assessment (MNA) or MNA-Short Form. These methods demand extended screening periods and lack specific biological factors (40, 44). In contrast, the GNRI offers a more straightforward approach, relying solely on serum albumin levels, height, and weight measurements for each individual. Prior studies have underscored the value of GNRI in evaluating the physical well-being of elderly patients with chronic illnesses (45). In our research, we found compelling evidence that the GNRI serves as a valuable and convenient predictive biomarker for survival outcomes in ICI-treated cancer patients.

Along with controlling osmotic pressure and transporting bioactive molecules, albumin, a GNRI component, is also recognized to have immunomodulatory properties. For instance, albumin prevents neutrophils from overreacting by inhibiting inflammation (46, 47). Albumin suppresses neutrophil extracellular trap formation in the tumor microenvironment, where neutrophils emit neutrophil extracellular traps, facilitating tumor development and metastasis (48–50). Furthermore, albumin possesses antioxidant capabilities and decreases oxidative stress in tissues (46, 47). Through altered cytokine signaling, increased immunosuppressive immune cell activity, and decreased cytotoxic lymphocytes, oxidative stress causes immunosuppression in the tumor microenvironment (51). It has been demonstrated that under oxidative stress, regulatory T cells cause significant immunosuppression, which eliminates the anticancer immunity response by PD-L1 inhibition *in vivo* (52). The immunomodulatory activity of albumin may favor tumor immunity in the tumor microenvironment.

Another element of GNRI, body weight, has drawn interest as a potential indicator of ICI effectiveness. As compared to control diet-fed mice, obese animals brought on by diet showed superior responses to anti-PD-1 therapy (52). It is believed that factors related to adipose tissue contribute to cancer immunity, despite the fact that the precise mechanisms underlying the increased efficacy of ICI therapy in obesity have not been elucidated (53). Furthermore, it has been demonstrated that improved survival outcomes in overweight patients can be attributed to the role of white adipose tissue as a source of cytokines and chemokines that induce and/or coordinate host defenses (54, 55). Adipose tissue can modulate the balance between helper T-cell (Th)1 and Th2 responses, downregulating regulatory T-cell activation through adiponectin, promoting the presence of pro-inflammatory macrophages, activating T-cells, and enhancing the inflammatory state through the CD40 pathway (56–58). Therefore, the preclinical studies mentioned above fully support the idea that high GNRI levels contribute to a better immune response.

Notably, in addition to PD-(L)1 and CTLA-4, TGF-β also promotes immune escape. In recent years, anti-TGF-β/PD-L1 bispecific antibodies such as YM101 and BiTP have been developed (59, 60). However, there are no studies examining the relationship between GNRI and the efficacy of anti-TGF-β/PD-L1 bispecific antibodies. Therefore, only cancer patients treated with PD-(L) or CTLA4 were included in this study, and the relationship between GNRI and anti-TGF-β/PD-L1 bispecific antibodies needs to be further investigated.

In conclusion, this study demonstrates that GNRI is an important prognostic biomarker for ICI-treated cancer patients. This simple classification may be useful in clinical practice. Our evidence of interrogative medicine needs to be validated by further external multicenter randomized controlled studies.

## Data availability statement

The original contributions presented in the study are included in the article/Supplementary material, further inquiries can be directed to the corresponding authors.

## Author contributions

LZ: Conceptualization, Formal analysis, Investigation, Methodology, Writing – original draft. KW: Conceptualization, Data curation, Formal analysis, Investigation, Writing – original draft. TK: Investigation, Software, Writing – original draft. WD: Supervision, Writing – review & editing. PH: Conceptualization, Methodology, Validation, Visualization, Writing – review & editing. WW: Investigation, Supervision, Validation, Writing – review & editing.

## References

[1] Gibney GT Weiner LM Atkins MB. Predictive biomarkers for checkpoint inhibitor-based immunotherapy. Lancet Oncol. (2016) 17:e542–51. doi: 10.1016/S1470-2045(16)30406-5, PMID: 27924752 PMC5702534

[2] Sharma P Allison JP. The future of immune checkpoint therapy. Science. (2015) 348:56–61. doi: 10.1126/science.aaa8172 25838373

[3] Liu J Zhong L Deng D Zhang Y Yuan Q Shang D. The combined signatures of the tumour microenvironment and nucleotide metabolism-related genes provide a prognostic and therapeutic biomarker for gastric cancer. Sci Rep. (2023) 13:6622. doi: 10.1038/s41598-023-33213-z, PMID: 37095256 PMC10126105

[4] Zhang B Yuan Q Zhang B Li S Wang Z Liu H . Characterization of neuroendocrine regulation- and metabolism-associated molecular features and prognostic indicators with aid to clinical chemotherapy and immunotherapy of patients with pancreatic cancer. Front Endocrinol (Lausanne). (2022) 13:1078424. doi: 10.3389/fendo.2022.1078424 36743929 PMC9895410

[5] Fancello L Gandini S Pelicci PG Mazzarella L. Tumor mutational burden quantification from targeted gene panels: major advancements and challenges. J Immunother Cancer. (2019) 7:183. doi: 10.1186/s40425-019-0647-4, PMID: 31307554 PMC6631597

[6] Wang Z Yuan Q Chen X Luo F Shi X Guo F . A prospective prognostic signature for pancreatic adenocarcinoma based on ubiquitination-related mRNA-lncRNA with experimental validation in vitro and vivo. Funct Integr Genomics. (2023) 23:263. doi: 10.1007/s10142-023-01158-1, PMID: 37540295 PMC10403435

[7] Reck M Rodríguez-Abreu D Robinson AG Hui R Csőszi T Fülöp A . Pembrolizumab versus chemotherapy for PD-L1-positive non-small-cell lung Cancer. N Engl J Med. (2016) 375:1823–33. doi: 10.1056/NEJMoa1606774 27718847

[8] Borghaei H Paz-Ares L Horn L Spigel DR Steins M Ready NE . Nivolumab versus docetaxel in advanced nonsquamous non-small-cell lung Cancer. N Engl J Med. (2015) 373:1627–39. doi: 10.1056/NEJMoa1507643, PMID: 26412456 PMC5705936

[9] Brahmer J Reckamp KL Baas P Crinò L Eberhardt WEE Poddubskaya E . Nivolumab versus docetaxel in advanced squamous-cell non-small-cell lung Cancer. N Engl J Med. (2015) 373:123–35. doi: 10.1056/NEJMoa1504627, PMID: 26028407 PMC4681400

[10] Mazieres J Rittmeyer A Gadgeel S Hida T Gandara DR Cortinovis DL . Atezolizumab versus docetaxel in pretreated patients with NSCLC: final results from the randomized phase 2 POPLAR and phase 3 OAK clinical trials. J Thorac Oncol. (2021) 16:140–50. doi: 10.1016/j.jtho.2020.09.022, PMID: 33166718

[11] Galmés S Serra F Palou A. Current state of evidence: influence of nutritional and Nutrigenetic factors on immunity in the COVID-19 pandemic framework. Nutrients. (2020) 12:2738. doi: 10.3390/nu12092738, PMID: 32911778 PMC7551697

[12] Faverio P de Giacomi F Bodini BD Stainer A Fumagalli A Bini F . Nontuberculous mycobacterial pulmonary disease: an integrated approach beyond antibiotics. ERJ Open Res. (2021) 7:00574–2020. doi: 10.1183/23120541.00574-2020, PMID: 34046491 PMC8141831

[13] Healy C Munoz-Wolf N Strydom J Faherty L Williams NC Kenny S . Nutritional immunity: the impact of metals on lung immune cells and the airway microbiome during chronic respiratory disease. Respir Res. (2021) 22:133. doi: 10.1186/s12931-021-01722-y, PMID: 33926483 PMC8082489

[14] Yamada K Furuya R Takita T Maruyama Y Yamaguchi Y Ohkawa S . Simplified nutritional screening tools for patients on maintenance hemodialysis. Am J Clin Nutr. (2008) 87:106–13. doi: 10.1093/ajcn/87.1.106, PMID: 18175743

[15] Matsumura T Mitani Y Oki Y Fujimoto Y Ohira M Kaneko H . Comparison of geriatric nutritional risk index scores on physical performance among elderly patients with chronic obstructive pulmonary disease. Heart Lung. (2015) 44:534–8. doi: 10.1016/j.hrtlng.2015.08.004, PMID: 26409897

[16] Matsuo Y Kumakura H Kanai H Iwasaki T Ichikawa S. The geriatric nutritional risk index predicts long-term survival and cardiovascular or limb events in peripheral arterial disease. J Atheroscler Thromb. (2020) 27:134–43. doi: 10.5551/jat.49767, PMID: 31217396 PMC7049470

[17] Wada H Dohi T Miyauchi K Doi S Naito R Konishi H . Prognostic impact of the geriatric nutritional risk index on long-term outcomes in patients who underwent percutaneous coronary intervention. Am J Cardiol. (2017) 119:1740–5. doi: 10.1016/j.amjcard.2017.02.051, PMID: 28388993

[18] Lee GW Go SI Kim DW Kim HG Kim JH An HJ . Geriatric nutritional risk index as a prognostic marker in patients with extensive-stage disease small cell lung cancer: results from a randomized controlled trial. Thorac Cancer. (2020) 11:62–71. doi: 10.1111/1759-7714.13229, PMID: 31707767 PMC6938749

[19] Okamoto T Hatakeyama S Narita S Takahashi M Sakurai T Kawamura S . Impact of nutritional status on the prognosis of patients with metastatic hormone-naïve prostate cancer: a multicenter retrospective cohort study in Japan. World J Urol. (2019) 37:1827–35. doi: 10.1007/s00345-018-2590-2, PMID: 30511214

[20] Migita K Matsumoto S Wakatsuki K Ito M Kunishige T Nakade H . The prognostic significance of the geriatric nutritional risk index in patients with esophageal squamous cell carcinoma. Nutr Cancer. (2018) 70:1237–45. doi: 10.1080/01635581.2018.1512640, PMID: 30235009

[21] Kanno H Goto Y Sasaki S Fukutomi S Hisaka T Fujita F . Geriatric nutritional risk index predicts prognosis in hepatocellular carcinoma after hepatectomy: a propensity score matching analysis. Sci Rep. (2021) 11:9038. doi: 10.1038/s41598-021-88254-z, PMID: 33907232 PMC8079680

[22] Tang QN Qiu HZ Sun XQ Guo SS Liu LT Wen YF . Geriatric nutritional risk index as an independent prognostic factor in locally advanced nasopharyngeal carcinoma treated using radical concurrent chemoradiotherapy: a retrospective cohort study. Ann Transl Med. (2021) 9:532. doi: 10.21037/atm-20-6493, PMID: 33987230 PMC8105839

[23] Chang LW Hung SC Li JR Chiu KY Yang CK Chen CS . Geriatric nutritional risk index as a prognostic marker for patients with metastatic castration-resistant prostate Cancer receiving docetaxel. Front Pharmacol. (2020) 11:601513. doi: 10.3389/fphar.2020.601513 33569000 PMC7868324

[24] Shoji F Matsubara T Kozuma Y Haratake N Akamine T Takamori S . Preoperative geriatric nutritional risk index: a predictive and prognostic factor in patients with pathological stage I non-small cell lung cancer. Surg Oncol. (2017) 26:483–8. doi: 10.1016/j.suronc.2017.09.006, PMID: 29113668

[25] Matsuura S Morikawa K Ito Y Kubota T Ichijo K Mochizuki E . The geriatric nutritional risk index and prognostic nutritional index predict the overall survival of advanced non-small cell lung cancer patients. Nutr Cancer. (2022) 74:1606–13. doi: 10.1080/01635581.2021.1960387, PMID: 34431441

[26] Sonehara K Tateishi K Araki T Komatsu M Yamamoto H Hanaoka M. Prognostic value of the geriatric nutritional risk index among patients with previously treated advanced non-small cell lung cancer who subsequently underwent immunotherapy. Thorac Cancer. (2021) 12:1366–72. doi: 10.1111/1759-7714.13909, PMID: 33710780 PMC8088948

[27] Liberati A Altman DG Tetzlaff J Mulrow C Gøtzsche PC Ioannidis JPA . The PRISMA statement for reporting systematic reviews and meta-analyses of studies that evaluate health care interventions: explanation and elaboration. PLoS Med. (2009) 6:e1000100. doi: 10.1371/journal.pmed.1000100, PMID: 19621070 PMC2707010

[28] Zhang L Chen C Chai D Li C Kuang T Liu L . Effects of PPIs use on clinical outcomes of urothelial cancer patients receiving immune checkpoint inhibitor therapy. Front Pharmacol. (2022) 13:1018411. doi: 10.3389/fphar.2022.1018411, PMID: 36225582 PMC9549125

[29] Zhang L Kuang T Chai D Deng W Wang P Wang W. The use of antibiotics during immune checkpoint inhibitor treatment is associated with lower survival in advanced Esophagogastric Cancer. Int Immunopharmacol. (2023) 119:110200. doi: 10.1016/j.intimp.2023.110200, PMID: 37099942

[30] Etani T Naiki T Sugiyama Y Nagai T Iida K Noda Y . Low geriatric nutritional risk index as a poor prognostic marker for second-line Pembrolizumab treatment in patients with metastatic urothelial carcinoma: a retrospective multicenter analysis. Oncology. (2020) 98:876–83. doi: 10.1159/000508923, PMID: 32862183

[31] Fujiwara R Yuasa T Yamamoto S Fujiwara M Takemura K Urasaki T . Geriatric nutritional risk index as a predictor of prognosis in metastatic renal cell carcinoma treated with Nivolumab. Nutr Cancer. (2023) 75:670–7. doi: 10.1080/01635581.2022.2152061, PMID: 36448767

[32] Haas M Lein A Fuereder T Brkic FF Schnoell J Liu DT . The geriatric nutritional risk index (GNRI) as a prognostic biomarker for immune checkpoint inhibitor response in recurrent and/or metastatic head and neck cancer. Nutrients. (2023) 15:880. doi: 10.3390/nu15040880, PMID: 36839241 PMC9961934

[33] Hiraoka A Kumada T Tada T Hirooka M Kariyama K Tani J . Geriatric nutritional risk index as an easy-to-use assessment tool for nutritional status in hepatocellular carcinoma treated with atezolizumab plus bevacizumab. Hepatol Res. (2023) 53:1031–42. doi: 10.1111/hepr.13934, PMID: 37306040

[34] Isobe T Naiki T Sugiyama Y Naiki-Ito A Nagai T Etani T . Chronological transition in outcome of second-line treatment in patients with metastatic urothelial cancer after pembrolizumab approval: a multicenter retrospective analysis. Int J Clin Oncol. (2022) 27:165–74. doi: 10.1007/s10147-021-02046-z, PMID: 34633579

[35] Karayama M Inoue Y Yoshimura K Hozumi H Suzuki Y Furuhashi K . Association of the geriatric nutritional risk index with the survival of patients with non-small cell lung cancer after Nivolumab therapy. J Immunother. (2022) 45:125–31. doi: 10.1097/CJI.0000000000000396, PMID: 34653100 PMC8806036

[36] Shimizu T Miyake M Hori S Ichikawa K Omori C Iemura Y . Clinical impact of sarcopenia and inflammatory/nutritional markers in patients with unresectable metastatic urothelial carcinoma treated with pembrolizumab. Diagnostics. (2020) 10:310. doi: 10.3390/diagnostics10050310, PMID: 32429323 PMC7277993

[37] Tanaka K Hirakawa H Suzuki M Higa T Agena S Hasegawa N . Biomarkers for predicting anti-programmed cell Death-1 antibody treatment effects in head and neck cancer. Curr Oncol. (2023) 30:5409–24. doi: 10.3390/curroncol30060410, PMID: 37366893 PMC10297315

[38] Zheng X Gu H Cao X Pan B Xiang H Ju M . Tislelizumab for cervical cancer: a retrospective study and analysis of correlative blood biomarkers. Front Immunol. (2023) 14:1113369. doi: 10.3389/fimmu.2023.1113369 36875089 PMC9975598

[39] Liu C Zhao H Wang P Guo Z Qu Z. The combination of circulating IgM and geriatric nutritional risk index predicts the prognostic of hepatocellular carcinoma patients who underwent immune checkpoint inhibitors. Int Immunopharmacol. (2023) 123:110704. doi: 10.1016/j.intimp.2023.110704, PMID: 37506504

[40] Dent E Chapman I Piantadosi C Visvanathan R. Nutritional screening tools and anthropometric measures associate with hospital discharge outcomes in older people. Australas J Ageing. (2015) 34:E1–6. doi: 10.1111/ajag.12130, PMID: 24444126

[41] Ding P Wu J Wu H Sun C Guo H Lowe S . Inflammation and nutritional status indicators as prognostic indicators for patients with locally advanced gastrointestinal stromal tumors treated with neoadjuvant imatinib. BMC Gastroenterol. (2023) 23:23. doi: 10.1186/s12876-023-02658-x, PMID: 36690935 PMC9869595

[42] Zhang Q Zhang L Jin Q He Y Wu M Peng H . The prognostic value of the GNRI in patients with stomach cancer undergoing surgery. J Pers Med. (2023) 13:155. doi: 10.3390/jpm13010155, PMID: 36675816 PMC9861269

[43] Ding P Wu H Liu P Sun C Yang P Tian Y . The inflammatory burden index: a promising prognostic predictor in patients with locally advanced gastric cancer. Clin Nutr. (2023) 42:247–8. doi: 10.1016/j.clnu.2023.01.005, PMID: 36653262

[44] Marshall S Young A Bauer J Isenring E. Nutrition screening in geriatric rehabilitation: criterion (concurrent and predictive) validity of the malnutrition screening tool and the mini nutritional assessment-short form. J Acad Nutr Diet. (2016) 116:795–801. doi: 10.1016/j.jand.2015.06.012, PMID: 26212448

[45] Komatsu M Okazaki M Tsuchiya K Kawaguchi H Nitta K. Geriatric nutritional risk index is a simple predictor of mortality in chronic hemodialysis patients. Blood Purif. (2015) 39:281–7. doi: 10.1159/000381798, PMID: 25925239

[46] Wiedermann CJ. Hypoalbuminemia as surrogate and culprit of infections. Int J Mol Sci. (2021) 22:4496. doi: 10.3390/ijms22094496, PMID: 33925831 PMC8123513

[47] Ferrer R Mateu X Maseda E Yébenes JC Aldecoa C de Haro C . Non-oncotic properties of albumin. A multidisciplinary vision about the implications for critically ill patients. Expert Rev Clin Pharmacol. (2018) 11:125–37. doi: 10.1080/17512433.2018.1412827, PMID: 29219627

[48] Erpenbeck L Schön MP. Neutrophil extracellular traps: protagonists of cancer progression? Oncogene. (2017) 36:2483–90. doi: 10.1038/onc.2016.406, PMID: 27941879

[49] Neubert E Senger-Sander SN Manzke VS Busse J Polo E Scheidmann SEF . Serum and serum albumin inhibit in vitro formation of neutrophil extracellular traps (NETs). Front Immunol. (2019) 10:12. doi: 10.3389/fimmu.2019.00012, PMID: 30733715 PMC6354573

[50] Ding P Lv J Sun C Chen S Yang P Tian Y . Combined systemic inflammatory immunity index and prognostic nutritional index scores as a screening marker for sarcopenia in patients with locally advanced gastric cancer. Front Nutr. (2022) 9:981533. doi: 10.3389/fnut.2022.981533, PMID: 36046129 PMC9421237

[51] Augustin RC Delgoffe GM Najjar YG. Characteristics of the tumor microenvironment that influence immune cell functions: hypoxia, oxidative stress, metabolic alterations. Cancers (Basel). (2020) 12:3802. doi: 10.3390/cancers12123802 33348579 PMC7765870

[52] Maj T Wang W Crespo J Zhang H Wang W Wei S . Oxidative stress controls regulatory T cell apoptosis and suppressor activity and PD-L1-blockade resistance in tumor. Nat Immunol. (2017) 18:1332–41. doi: 10.1038/ni.3868, PMID: 29083399 PMC5770150

[53] Wang Z Aguilar EG Luna JI Dunai C Khuat LT le CT . Paradoxical effects of obesity on T cell function during tumor progression and PD-1 checkpoint blockade. Nat Med. (2019) 25:141–51. doi: 10.1038/s41591-018-0221-5, PMID: 30420753 PMC6324991

[54] Ouchi N Parker JL Lugus JJ Walsh K. Adipokines in inflammation and metabolic disease. Nat Rev Immunol. (2011) 11:85–97. doi: 10.1038/nri2921, PMID: 21252989 PMC3518031

[55] Xiao J Huang K Lin H Xia Z Zhang J Li D . Mogroside II(E) inhibits digestive enzymes via suppression of interleukin 9/interleukin 9 receptor signalling in acute pancreatitis. Front Pharmacol. (2020) 11:859. doi: 10.3389/fphar.2020.00859, PMID: 32587518 PMC7298197

[56] Jordan BF Gourgue F Cani PD. Adipose tissue metabolism and cancer progression: novel insights from gut microbiota? Curr Pathobiol Rep. (2017) 5:315–22. doi: 10.1007/s40139-017-0154-6, PMID: 29188139 PMC5684272

[57] Magrone T Jirillo E. Childhood obesity: immune response and nutritional approaches. Front Immunol. (2015) 6:76. doi: 10.3389/fimmu.2015.00076 25759691 PMC4338791

[58] Seijkens T Kusters P Chatzigeorgiou A Chavakis T Lutgens E. Immune cell crosstalk in obesity: a key role for costimulation? Diabetes. (2014) 63:3982–91. doi: 10.2337/db14-0272, PMID: 25414012

[59] Yi M Wu Y Niu M Zhu S Zhang J Yan Y . Anti-TGF-β/PD-L1 bispecific antibody promotes T cell infiltration and exhibits enhanced antitumor activity in triple-negative breast cancer. J Immunother Cancer. (2022) 10:e005543. doi: 10.1136/jitc-2022-005543, PMID: 36460337 PMC9723957

[60] Yi M Zhang J Li A Niu M Yan Y Jiao Y . The construction, expression, and enhanced anti-tumor activity of YM101: a bispecific antibody simultaneously targeting TGF-β and PD-L1. J Hematol Oncol. (2021) 14:27. doi: 10.1186/s13045-021-01045-x, PMID: 33593403 PMC7885589

