# Supplementary material for: Low geriatric nutritional risk index as a poor prognostic biomarker for immune checkpoint inhibitor treatment in solid cancer
Source: Front Nutr. 2023 Nov 1;10:1286583. doi: 10.3389/fnut.2023.1286583 (PMC10646500; doi:10.3389/fnut.2023.1286583)
Supplement: Supplementary file 1 [file Data_Sheet_1.docx]

Supplementary material 1. The detailed search strategies for Pubmed

((((((Camrelizumab) OR (Sintilimab)) OR (Tislelizumab)) OR (Toripalimab)) OR (Envafolimab)) OR (((((((((((((((((((((((((((((((((((((((((((((((((anti-CTLA4 antibody) OR (anti-CTLA4 antibodies)) OR (Immune Checkpoint Inhibitors)) OR (Checkpoint Inhibitors, Immune)) OR (Immune Checkpoint Inhibitor)) OR (Checkpoint Inhibitor, Immune)) OR (Immune Checkpoint Blockers)) OR (Checkpoint Blockers, Immune)) OR (Immune Checkpoint Blockade)) OR (Checkpoint Blockade, Immune)) OR (Immune Checkpoint Inhibition)) OR (Checkpoint Inhibition, Immune)) OR (PD-L1 Inhibitors)) OR (PD L1 Inhibitors)) OR (PD-L1 Inhibitor)) OR (PD L1 Inhibitor)) OR (Programmed Death-Ligand 1 Inhibitors)) OR (Programmed Death Ligand 1 Inhibitors)) OR (PD-1-PD-L1 Blockade)) OR (Blockade, PD-1-PD-L1)) OR (PD 1 PD L1 Blockade)) OR (CTLA-4 Inhibitors)) OR (CTLA 4 Inhibitors)) OR (CTLA-4 Inhibitor)) OR (CTLA 4 Inhibitor)) OR (Cytotoxic T-Lymphocyte-Associated Protein 4 Inhibitors)) OR (Cytotoxic T Lymphocyte Associated Protein 4 Inhibitors)) OR (Cytotoxic T-Lymphocyte-Associated Protein 4 Inhibitor)) OR (Cytotoxic T Lymphocyte Associated Protein 4 Inhibitor)) OR (PD-1 Inhibitors)) OR (PD-1 Inhibitor)) OR (PD 1 Inhibitors)) OR (Inhibitor, PD-1)) OR (PD 1 Inhibitor)) OR (Programmed Cell Death Protein 1 Inhibitor)) OR (Programmed Cell Death Protein 1 Inhibitors)) OR (pembrolizumab)) OR (nivolumab)) OR (atezolizumab)) OR (ipilimumab)) OR (avelumab)) OR (tremelimumab)) OR (durvalumab)) OR (cemiplimab)) OR (anti-PD-1 antibodies)) OR (anti-PD-1 antibody)) OR (anti-PD-L1 antibody)) OR (anti-PD-L1 antibodies)) OR (Immune Checkpoint Inhibitors[MeSH Terms]))) AND ((Geriatric Nutritional Risk Index) OR (GNRI))
